# Assay of Secondary Anisotropy in Additively Manufactured Alloys for Dental Applications

**DOI:** 10.3390/ma11101831

**Published:** 2018-09-26

**Authors:** Elena Bassoli, Lucia Denti

**Affiliations:** Department of Engineering “Enzo Ferrari”, University of Modena and Reggio Emilia, via Pietro Vivarelli 10, 41125 Modena, Italy; lucia.denti@unimore.it

**Keywords:** Powder Bed Fusion, Titanium alloys, Cobalt–Chrome alloys, anisotropy

## Abstract

Even though additive manufacturing (AM) techniques have been available since the late 1980s, their application in medicine is still striving to gain full acceptance. For the production of dental implants, the use of AM allows to save time and costs, but also to ensure closer dimensional tolerances and higher repeatability, as compared to traditional manual processes. Among the several AM solutions, Laser Powder Bed Fusion (L-PBF) is the most appropriate for the production of metal prostheses. The target of this paper was to investigate the mechanical and microstructural characteristics of Co–Cr–Mo and Ti–6Al–4V alloys processed by L-PBF, with a specific focus on secondary anisotropy that is usually disregarded in the literature. Tensile specimens were built in the EOSINT-M270 machine, along different orientations perpendicular to the growth direction. Density, hardness, and tensile properties were measured and the results combined with microstructural and fractographic examination. For both alloys, the results provided evidence of high strength and hardness, combined with outstanding elongation and full densification. Extremely fine microstructures were observed, sufficient to account for the good mechanical response. Statistical analysis of the mechanical properties allowed to attest the substantial absence of secondary anisotropy. The result was corroborated by the observations of the microstructures and of the failure modes. Overall, the two alloys proved to be high-performing, in very close agreement with the values reported in the datasheets, independently of the build orientation.

## 1. Introduction

Additive Manufacturing (AM) techniques allow the production of objects with complex geometry. Fabrication can be started straightforwardly by using a three-dimensional Computer Aided Design (CAD) model, without tools. The basic idea is to think of every object as consisting of thin layers, usually in the range of 0.03–0.05 mm. The part is built up by progressive addition of material, which enables unprecedented ease of manufacturing of extremely complex shapes, since the three-dimensional manufacturing issues are simplified to two-dimensional problems [1,2]. It becomes, thus, possible to produce parts with cavities and undercuts that, by conventional subtractive methods, would have been unfeasible or would have caused great manufacturing hurdles and costs. AM technologies were introduced in industry in the late 1980s to realize models and prototypes, but nowadays the advances in materials and technology are sufficient to make the production of end products of major interest [3,4]. The great potential and good evolution of techniques led to introduce AM in medicine, where the need for parts that are customized for each patient, with a high degree of personalization, allows full exploitation of the inherent benefits of additive processes [5]. In particular, the Laser-based Powder Bed Fusion (L-PBF) process can be successfully used for the production of prostheses [6], including for example long-span and cantilever metal-ceramic-fixed partial dentures for maxillary and mandibular prosthodontics [7,8]. Apart from restorations, also surgical guides aimed at operation planning can take advantage of the quick production by AM [6,9].

This paper focuses on the characterization of alloys developed on purpose for maxillo-facial surgery, in particular for oral implants. Implantology is a field under continuous innovation, where research efforts are concurrently dedicated to finding new materials, new components, new fabrication processes, with the aim to improve the duration, the aesthetics and the functionality of prostheses and thus ensuring a better quality of life for patients. An easy example is that of gold crowns that have been superseded by metal-ceramic or ceramic crowns [10,11,12]. An increasing variety of metallic biomaterials is being developed, ranging from commercially pure Titanium [13] and Titanium alloys, through stainless steels, to Cobalt–Chrome alloys. Promising results have recently been attained for innovative β-type Ti alloys with increased wear resistance and lower elastic modulus, so as to better match that of the human bone and prevent the stress shielding effect [14]. Some of these novel alloys exhibit composite microstructures where β–Ti dendrites are surrounded by intermetallic phases so that efficient reinforcing phenomena are established [15]. The promising outlook of these Titanium-based matrix composites for AM has been very recently reviewed [16].

L-PBF process can be used in the construction of metal-ceramic fixed partial dentures (FPDs). Traditionally, the manufacture of the metallic part of FPDs involves a large series of manual operations performed by the dental technician, and the result is often strongly determined by his ability. For metal-prostheses fabrication, the adoption of L-PBF grants a much higher repeatability and predictability with respect to the manual process [17]. Compared to other powder-based methods that require molds, PBF offers outstanding personalization capabilities, in extremely short times and with low costs. On the other hand, the L-PBF process is quite complex and many factors are involved in order to achieve good part quality [18]. Despite the extremely diffused studies on this process, many efforts are still needed to better understand the relation between microstructure, processing, and properties for parts built by L-PBF [19,20,21].

In L-PBF, at each step, a thin layer of metallic powder is evenly distributed onto the previous layer and a laser selectively scans the regions corresponding to the cross-section of the part. As a consequence, the powder melts and then consolidates into a solid slice. Inherent in the process are two types of possible anisotropy: a primary one, due to the superimposition of layers in the direction that is usually called Z; and a secondary one that may manifest as direction-dependence of properties even within the XY plane, that is to say parallel to the layers [22]. The latter is usually ignored by machine- and material suppliers, and has been disregarded by scientific literature until now. Secondary anisotropy may be caused for example by the action of the recoater blade that spreads the powder in the bed, or by the inert gas flux that blows the melting slags away from the build area [23]. Each of the two phenomena usually acts along either the X or the Y direction, depending on the specific machine architecture. The investigation of secondary anisotropy is markedly important if the intended application is the production of FPDs, because complete prostheses are not straight structures but develop along the maxillary/mandibular arch, hence they involve material properties in several directions of the XY plane. A robust design of the restoration requires a reliable knowledge of any direction-dependent feature.

This research tackles the mechanical properties and the microstructure of two L-PBF fabricated dental alloys, namely Co–Cr–Mo and Ti–6Al–4V, by proposing a statistically-based enquiry of secondary anisotropy.

## 2. Materials and Methods

Tensile specimens were produced by L-PBF using the two alloys Ti–6Al–4V (EOS GmbH, Krailling, Germany) and Co–Cr–Mo (EOS Cobalt Chrome MP1, EOS GmbH, Krailling, Germany).

The specimens were fabricated on the L-PBF machine EOSINT-M270, by using the following process parameters:

- for Ti–6Al–4V: laser power 340 W, laser spot diameter 0.1 mm, layer thickness 30 µm, scan speed 1250 mm/s, hatch distance 0.12 mm, protective atmosphere (max 0.1% oxygen);
- for Co–Cr–Mo: laser power 200 W, laser spot diameter 0.2 mm, layer thickness 20 µm, scan speed 7000 mm/s, hatch distance 0.3 mm, protective atmosphere (max 1.5% oxygen).

For both alloys, tensile specimens were built in three different orientations relative to the machine distinctive directions, all of the three parallel to the layers and perpendicular to the growth direction. The three groups, each of 6 specimens, are specified as follows:

- “X” group: the axis of the specimens aligns to the direction which the recoater blade spreads the powder in the bed along;
- “Y” group: the axis of the specimens aligns to the direction of the inert gas flux on the powder bed;
- “XY” group: the axis of the specimens is angled 45° with respect that of X and Y groups.

**Figure 1:**
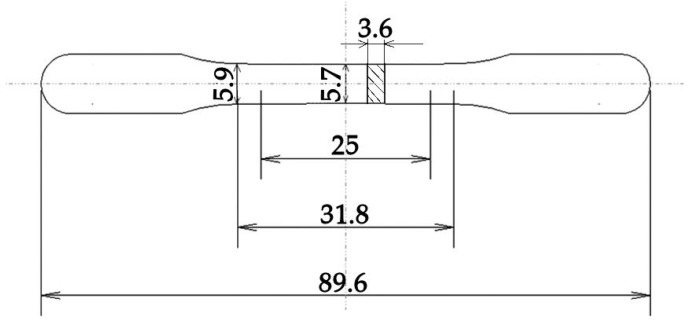
Tensile specimen. Dimensions are expressed in millimeters.

The size and geometry for the tensile test specimens conformed to the prescription specified in standard ASTM E8M [24]. Details are reported in Figure 1.

Of the powders, the nominal physical/mechanical properties and chemical composition are listed in Table 1 and Table 2, respectively. The powders were characterized by means of laser granulometry (Malvern Mastersizer 3000, Malvern Panalytical Ltd., Malvern, UK) to assess their size distribution, according to ISO 13320 standard [25].

The specimens were tested in the as built condition, without any heat treatment, so as to avoid any smoothing of the secondary anisotropy produced by the L-PBF process.

Before the tensile tests, the Archimedes principle was used to measure the density of all the samples (6 for each group), with an analytical electronic balance having a resolution of 0.1 mg (Pioneer^®^ Plus PA124C, OHAUS GmbH, Greifensee, Switzerland). The residual porosity was then calculated by using the nominal density of each alloy.

Tensile tests were performed on a SCHENK HYDROPULS PSB testing machine (SCHENCK RoTec GmbH, Darmstadt, Germany) with a capacity of 250 kN, using a crosshead speed of 5 mm/min. Five samples were tested for each alloy and orientation, and one extra specimen of each group was used to measure hardness and to obtain the metallographic sections. The choice of the hardness scale was made according to ISO standard 4498 [28]. Rockwell C was selected and performed following the specifications of standard ISO 6508 [29], by repeating five measurements on each sample. Numerical results for hardness (HRC), tensile strength (UTS) and total extension at fracture (ε_b_) were processed through statistical tools (Statistica 8, Statsoft, Hamburg, Germany): the *t*-test with a level of significance of 0.05 was performed to investigate the presence of significant differences between the groups of specimens produced along different orientations.

After tensile tests, rupture surfaces were observed by using a scanning electron microscope, SEM (ESEM, Quanta FEI, Thermo Fisher Scientific, Eindhoven, The Netherlands), in order to investigate the failure mechanisms and the joining phenomena between the particles.

Metallographic sections of the samples were obtained and observed by an optical microscope (OM) (Eclipse LV150N, Nikon, Tokyo, Japan), to get a cross-check of residual porosity and compare the results with those obtained by the Archimedes method. A comparative assessment of the two methods is raising the interests of the scientific community [30], growingly as the two techniques are more and more diffused in industry for the control of AM parts. Preparation of the metallographic sections consisted of micro-cutting, embedding in epoxy resin and polishing till a fine grinding. The final step was carried out with a plan cloth and 1 μm diamond suspension. Several micrographs were acquired through a CCD camera, made binary and analyzed through a software tool for image analysis to determine:

- percentage porosity, calculated as the area fraction of pores out of the overall area;
- the average pore area.

After OM observation, polished sections of Ti–6Al–4V underwent chemical etching with the Dix-Keller reactant (HF 2% vol, HCl 1.5% vol, HNO_3_ 2.5% vol; water bal.); while metallographic sections of the Co–Cr–Mo alloy were subjected to electrochemical etching (HCl 0.1 M, 2 V, 2 min). Microstructures were observed on the etched samples by means of OM and SEM.

## 3. Results and Discussion

### 3.1. Powder Particle Size Distribution

**Figure 2:**
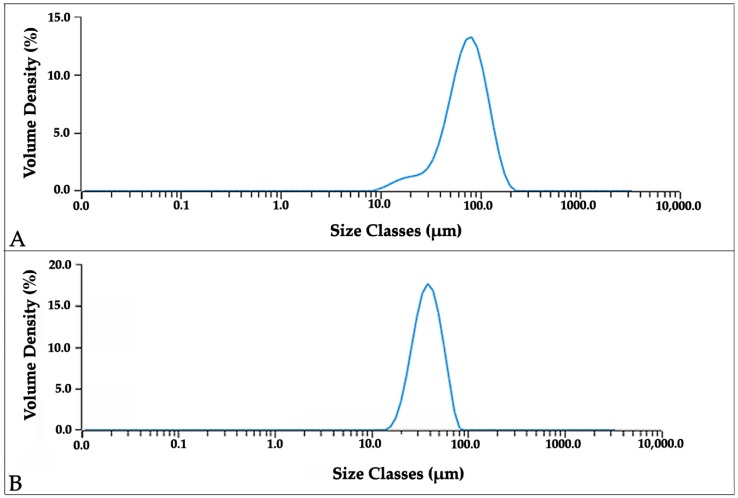
Particle size distribution of the Co–Cr–Mo (**A**) and Ti–6Al–4V (**B**) powders.

The results of laser granulometry of the two powders are shown in Figure 2. Co–Cr–Mo powder displays a wider distribution, with an average value of the order of 80 µm and a relatively large number of particles in the range 10–30 µm causing a negative skew in the curve. The size distribution for Ti–6Al–4V powder is instead symmetrical, with an average particle dimension of 30 µm.

### 3.2. Density and Residual Porosity

**Figure 3:**
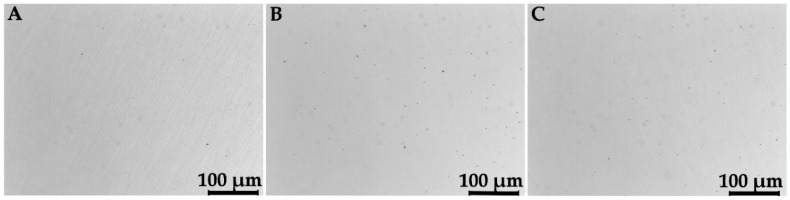
OM images of metallographic sections of Ti–6Al–4V specimens of the X (**A**), Y (**B**), and XY (**C**) groups.

**Figure 4:**
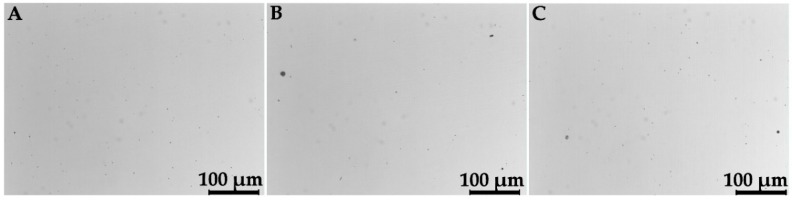
OM images of metallographic sections of Co–Cr–Mo specimens of the X (**A**), Y (**B**), and XY (**C**) groups.

**Figure 5:**
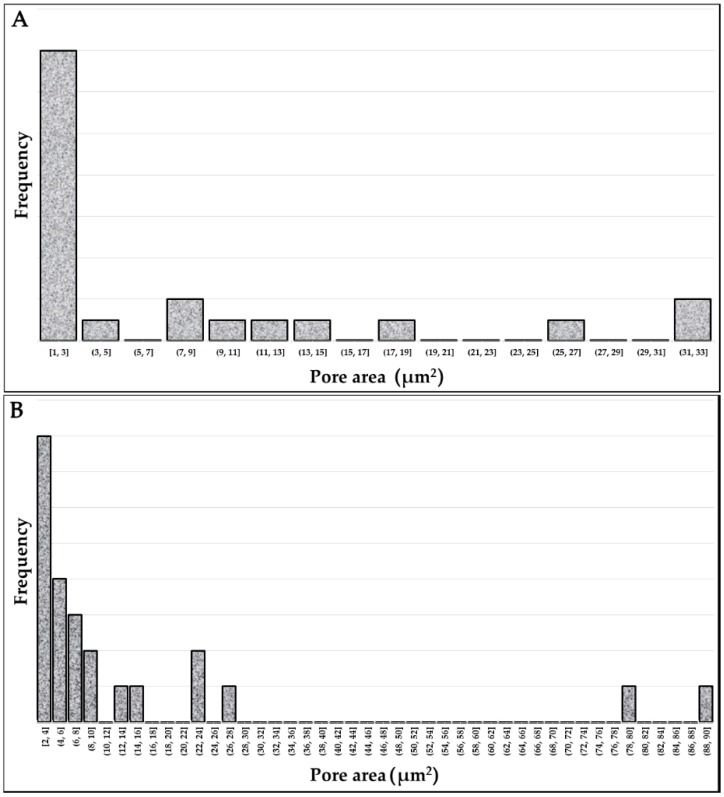
Distribution of the area of pores detected by the analysis of OM images: (**A**) Ti–6Al–4V, (**B**) Co–Cr–Mo.

Table 3 lists the results for density and for residual porosity measured by the Archimedes principle, as well as by OM observations of metallographic sections. Figure 3 and Figure 4 show, respectively for Ti–6Al–4V and Co–Cr–Mo, examples of the OM images on which residual porosity was calculated by image analysis. The nominal densities of the two alloys are available in Table 1 for comparison. While the data obtained by the Archimedes method are normally distributed, the data by microscopic analysis exhibit the asymmetrical distributions shown in Figure 5. Hence, mean values and standard deviations are listed in Table 4 for the Archimedes figures, whereas median and mean values are reported for the analysis of OM images. Density figures are, for both alloys, very close to the nominal values, with extremely narrow deviations and no evident direction dependence, as the differences between the values calculated for the X, Y, and XY groups are contained within the standard deviations. Residual porosity is in all cases well below 1%, with no distinction for the various orientations. If porosity is calculated by comparing the Archimedes density with the nominal one, the values are slightly higher than those obtained by metallographic observations, with the only exception of Ti–6Al–4V X specimens. Based on these results, the Archimedes method can be reckoned conservative if applied to density control of L-PBF fabricated parts. This remark is in very good agreement with the results attained by Spierings et al. [30], who found the Archimedes measurement highly accurate and repeatable for the control of metal parts produced by PBF. The same study also concludes that, in contrast, microscopic analysis of cross sections can give inconsistent values of density, with variations of up to 4% in the direction of an underestimate of the residual porosity. The two methods are found comparable by Spierings et al. only for low porosities.

### 3.3. Hardness and Tensile Tests

**Figure 6:**
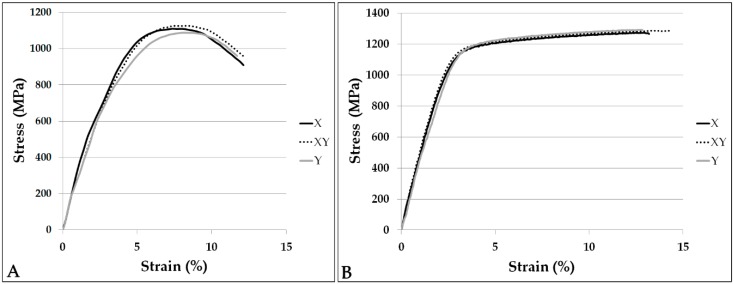
Example stress–strain curves of the Ti–6Al–4V (**A**) and Co–Cr–Mo (**B**) specimens built in the three orientations.

The results of hardness and tensile tests are listed in Table 4. Representative tensile stress–strain curves are shown in Figure 6. Necking is observed for Ti–6Al–4V, whereas the Co–Cr–Mo graphs are bilinear. As a term of comparison, mechanical properties reported in literature for the same alloys are given in Table 5. Normality of data distribution was verified by using the Shapiro-Wilk test, for all the mechanical characteristics recorded in Table 4. Then, the *t*-test was performed by grouping the mechanical properties according to the variable “orientation”. Table 6 registers the results, expressed in terms of probability values (*p*-values). When lower than 0.05, the *p*-values can be taken as a decision to reject the null hypothesis of absence of significant differences between the groups, that is to say of absence of anisotropy. In other terms, when the *p*-value is lower than 0.05 the mechanical response in different orientations can be regarded as non-equivalent.

HRC hardness is nearly 39 for Titanium alloy samples and 47 for Co–Cr–Mo specimens, with no statistically-significant differences between samples produced in different orientations. Mean UTS of Titanium specimens resulted in the range 1080–1110 MPa, and extension is about 12.5%. As for the Co–Cr–Mo alloy, mean UTS is between 1280 and 1300 MPa and values of 13–14%were obtained for total extension at fracture. All groups exhibit good test repeatability, with very low standard deviations, except for Ti–6Al–4V XY samples that show a relatively high scattering of the measured UTS. A slight anisotropy can be noticed for Ti–6Al–4V, consisting of a decreasing trend of strength from the X- towards the Y-orientation. Nevertheless, the variation is proportionately low (2.8%) and is pointed out as significant by the *t*-test only when two extreme groups, X and Y, are compared. A similar consideration is valid for Co–Cr–Mo samples, but in this case the opposite trend is observed: strength increases as the build orientation varies from the X- to the Y-direction. Even if the t-test is positive in two of the three cases, yet the overall deviation is as little as 1.6%. From a practical standpoint, for any industrial application the mechanical properties in the three directions would be considered undifferentiated, as a deviation of few per cents is by far absorbed by the factor of safety. As to ductility, for all the specimens the values are remarkably high if compared either to the nominal characteristics or to the typical properties of L-PBF fabricated parts. Furthermore, no direction dependence is evidenced for total extension at fracture.

### 3.4. Fractography

**Figure 7:**
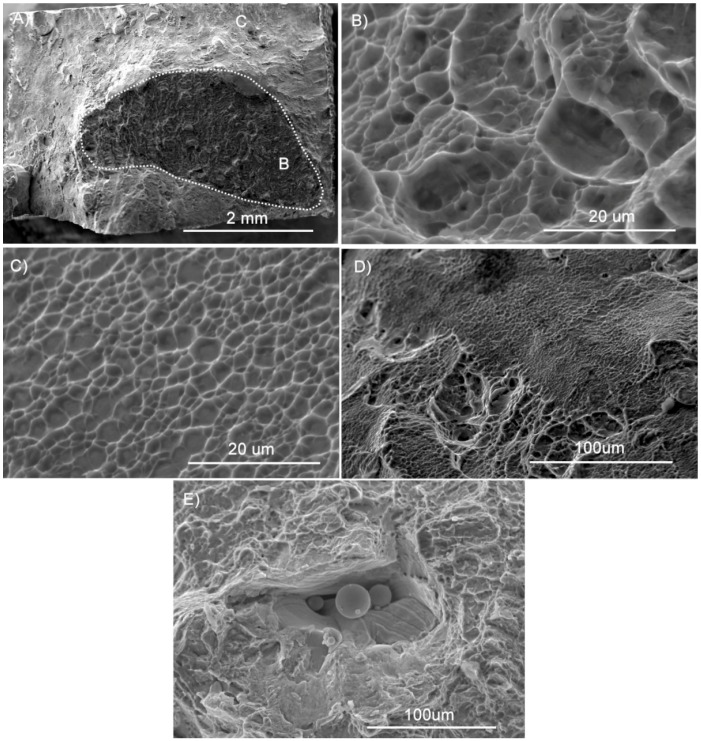
SEM observations of the rupture surface of a Ti–6Al–4V X specimen: (**A**) overall view; (**B**) dimpled rupture morphology; (**C**) quasi-flat rupture morphology; (**D**) mixed morphology; (**E**) lack of fusion defect.

**Figure 8:**
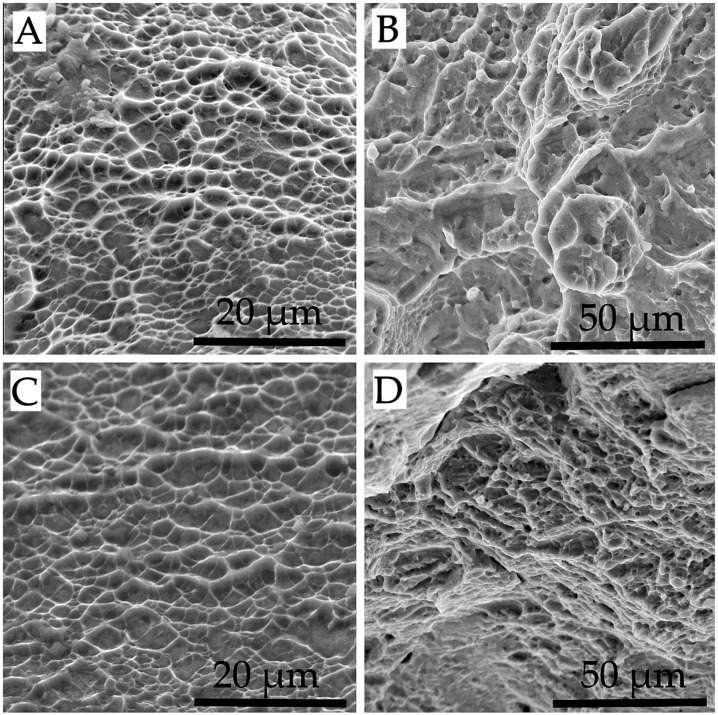
SEM observations of the rupture surfaces of a Ti–6Al–4V Y (**A**,**B**) and XY (**C**,**D**): (**A**,**C**) quasi-flat rupture morphology; (**B**,**D**) dimpled rupture morphology.

For Ti–6Al–4V specimens, failure occurs by a variety of mechanisms that can be observed comprehensively on macro-views of the rupture surfaces, as in the example in Figure 7A. The rupture shown is a typical failure mode of ductile materials, usually designated as cup and cone rupture. This form of ductile failure begins after necking and develops through sequential steps. At the outset, small micro-voids appear in the innermost zone of the specimen. Then, as plastic deformation proceeds, the micro-voids expand and merge into a crack. Figure 7B,C allow to appreciate, at high magnifications, the failure morphologies of the areas that are marked in Figure 7A as “B” and “C”, respectively. As to the first, laterally-fine but raised dimples can be observed, with a large amount of plastic deformation. This morphology can also be designated as cellular fracture and is often detected on the rupture surfaces of additively manufactured multi-phase materials [36]. On the last-breaking areas, as in Figure 7C, dimples are much flatter, indicating a low energy dissipation. Figure 7D shows a zone of transition between the two described failure modes. Lack of fusion defects are exceptionally spotted on the rupture surfaces (Figure 7E) [24]. Failure modes and rupture morphologies are identical for the specimens produced in the three orientations, as can be reckoned by comparing Figure 7 (X specimen) with Figure 8 (Y and XY specimens).

**Figure 9:**
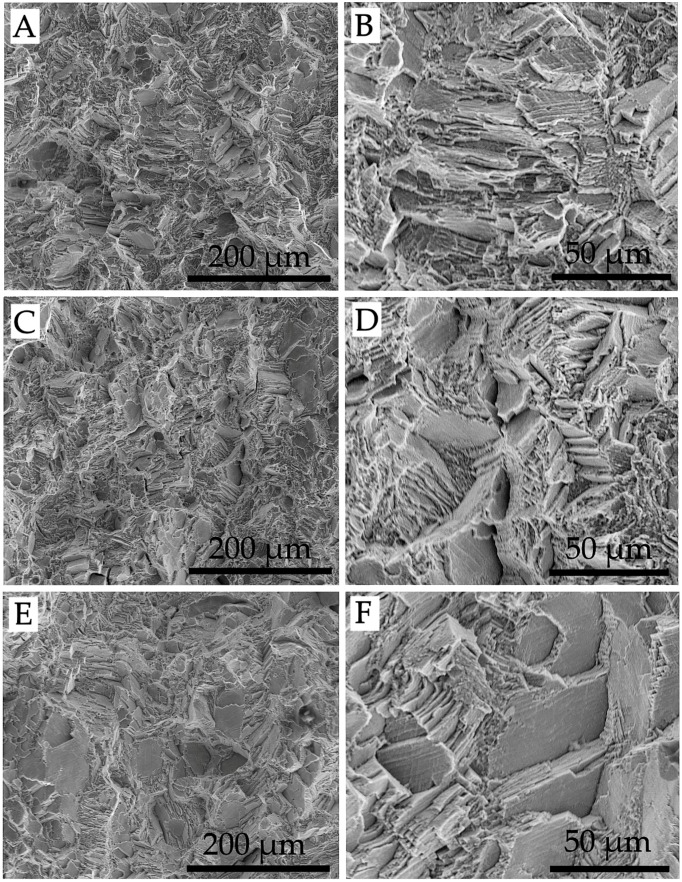
SEM observations of the rupture surface of Co–Cr–Mo specimens: (**A**,**B**) X; (**C**,**D**) Y; (**E**,**F**) XY.

Co–Cr–Mo specimens exhibit a homogeneous morphology across the rupture surfaces. A representative case for each of the three build orientations is shown in Figure 9, where failure seems to occur mainly by transgranular cleavage, even if the values of extension at fracture would suggest a more ductile mode. In these cases, the term “quasi-cleavage” is usually adopted, to identify a rupture that combines cleavage-like features with evidence of plastic deformation. Also, in this case, as for Ti–6Al–4V, the rupture surfaces of the three groups of samples are totally equal to each other.

For both alloys, if the different building orientations are compared, different rupture morphologies can be perceived in the macroscale, but the micro-mechanisms are in effect equal. This result is consistent with the substantial equivalence of the mechanical properties that has been discussed in Section 3.3. Overall, fractography suggests an extremely fine grain structure, which will be verified in the next section by means of observations of etched sections.

### 3.5. Microstructure

**Figure 10:**
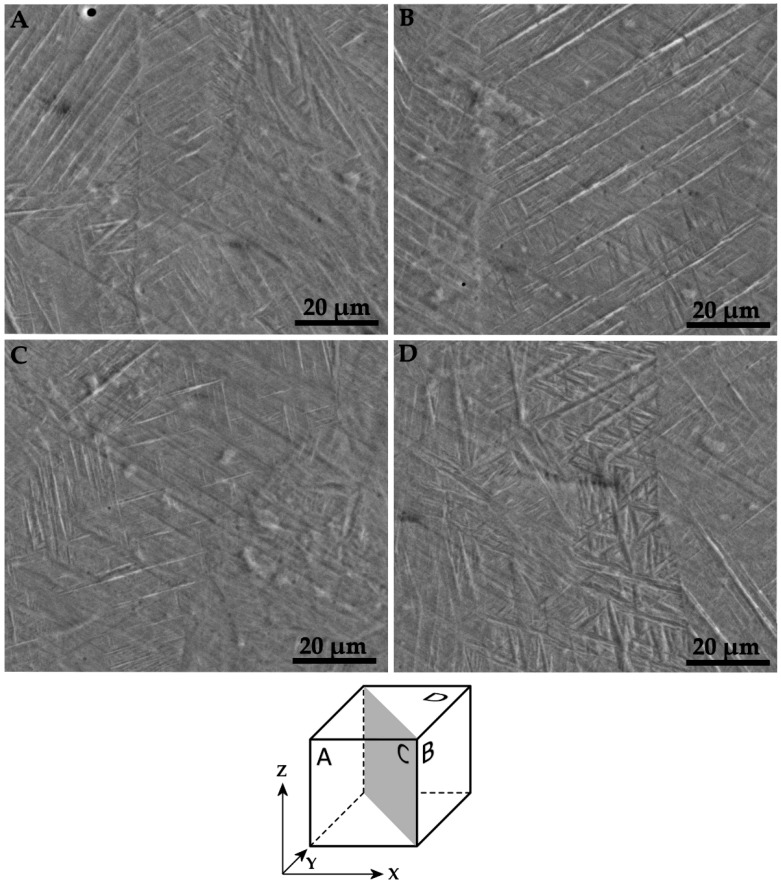
SEM images of etched sections of Ti–6Al–4V specimens. Alignment of the (**A**–**D**) images is schematized in the cube.

The microstructure observed for Ti–6Al–4V samples in the different orientations is visible in Figure 10. The sections in Figure 9B,C and Figure 10A are perpendicular to the axes of a Y, X, and XY specimen, respectively. Figure 10D allows to appreciate the microstructure in the XY plane. The Ti–6Al–4V specimens exhibit a uniform acicular α′ martensite microstructure [13], which forms under elevated cooling rates. Rapid cooling is frequently reported in literature for AM processes [19,37,38]. As an example, Criales et al. in a study on the L-PBF process of Inconel 625 measured cooling rates in the order of 150 °C/ms [38]. For Ti–6Al–4V, a rapid quenching is known in literature to cause a martensitic transformation, leading to a very fine needle-like microstructure [39]. No evidence of direction-dependent features is noticed.

**Figure 11:**
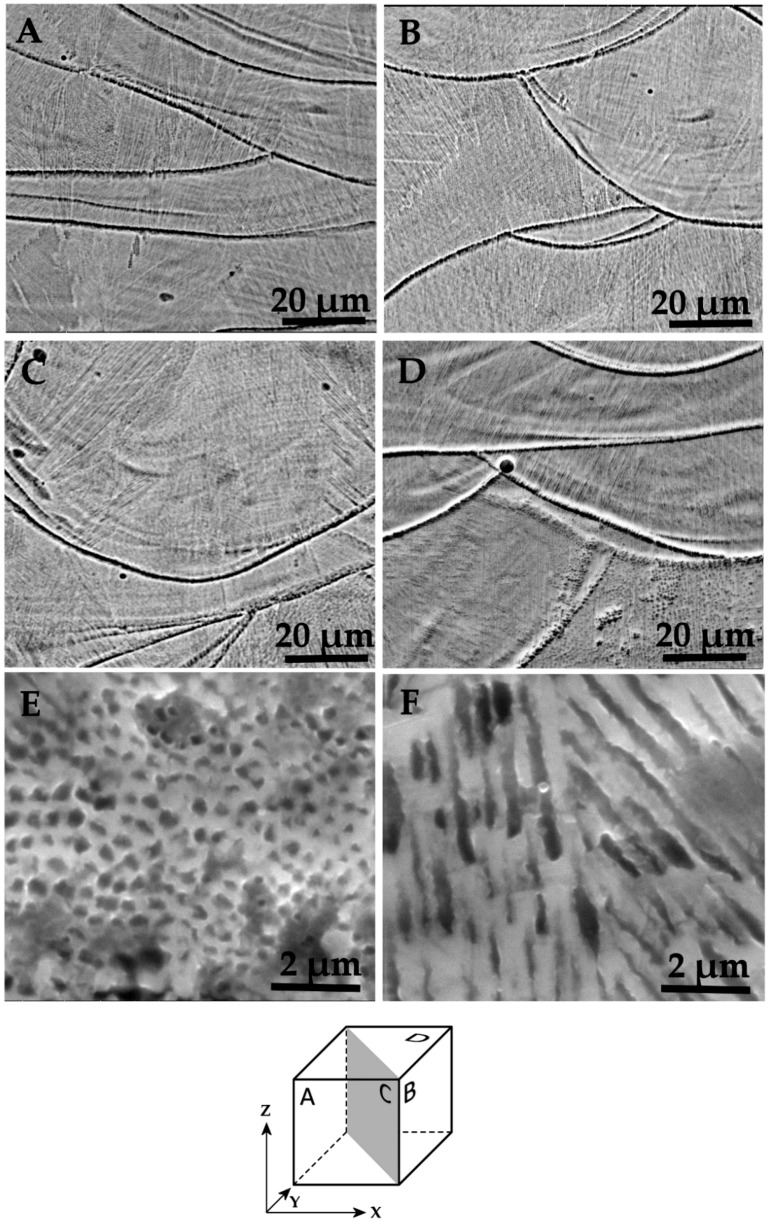
SEM images of etched sections of Co–Cr–Mo specimens. Alignment of the (**A**–**D**) images is schematized in the cube. (**E**,**F**) Detail of the columnar grains.

Images of the etched sections of Co–Cr–Mo specimens, referred to all the orientations already shown for Ti–6Al–4V samples, are provided in Figure 11A,D. For this alloy, etching reveals the boundaries of the melt pools. In all directions, within each melt pool an ultra-fine columnar microstructure can be appreciated at high magnification (Figure 11E,F), with varying grain orientation. The columnar grains have sub-micron diameter. No evidence of a martensitic structure is visible in Figure 11, however these observations are not conclusive on the point, in the absence of specific investigations, as for example X-ray diffractions.

## 4. Conclusions

In view of the increasing interest in the use of additively manufactured parts for dental prostheses, the mechanical behavior and the microstructure of Ti–6Al–4V and Co–Cr–Mo parts, built by L-PBF, were investigated, with a specific focus on the evaluation of secondary anisotropy.

For both alloys, the measured hardness and strength were in good agreement with those reported in the datasheets; ductility was remarkably high and nearly full densification was measured. The observed microstructures, typical of the extreme cooling rates experienced by the materials during L-PBF processes, allow to account for the outstanding mechanical properties that were appraised in this study. Statistical analysis of the mechanical properties allowed to attest the substantial absence of secondary anisotropy and the result was confirmed by the observation of identical failure modes of the specimens produced in the different orientations.

On the whole, the results enable the conclusion that the two alloys considered here may achieve exceptionally high properties if manufactured by L-PBF, and that secondary anisotropy is negligible if not totally absent.

## Figures and Tables

**Table 1:** Nominal physical and mechanical properties of the two alloys.

Property	Ti–6Al–4V [26]	Co–Cr–Mo [27]
Melting point (°C)	1634–1664	1350–1430
Density (kg/dm^3^)	4.43	8.3
Tensile strength (MPa) *	1150	1350
Total extension at fracture (%) *	11	11

* in horizontal direction, as built condition.

**Table 2:** Nominal chemical composition of the two alloys.

Alloy	Weight Percentage						
Ti–6Al–4V [26]	Ti	Al	V	C	Fe	N	O
-	88–90.2	5.5–6.8	3.5–4–5	<0.08	<0.30	<0.05	<0.2
Co–Cr–Mo [27]	Co	Cr	Mo	Si	Mn	other	
-	59.5	31.5	5.0	2.0	1.0	1.0	

**Table 3:** Density and porosity determined by the Archimedes principle and by OM observations. For Archimedes measurements, standard deviations are given in brackets next to the mean values computed over 6 measurements. For the results obtained by OM, median and mean values are provided.

Density and Porosity	Ti–6Al–4V		Co–Cr–Mo	
Archimedes	density (kg/dm^3^) mean (SD)	residual porosity (%) mean	density (kg/dm^3^) mean (SD)	residual porosity (%) mean
X	4.43 (0.00)	0	8.24 (0.01)	0.72
XY	4.41 (0.02)	0.45	8.26 (0.01)	0.48
Y	4.40 (0.02)	0.68	8.25 (0.06)	0.60
Analysis of OM images	average pore area (µm^2^)	residual porosity (%)	average pore area (µm^2^)	residual porosity (%)
	median–mean	median–mean	median–mean	median–mean
X	1.73–4.58	0.00–0.04	5.45–15.87	0.31–0.55
XY	1.82–2.04	0.19–0.21	5.03–10.09	0.17–0.30
Y	13.67–17.60	0.21–0.28	7.76–18.78	0.25–0.43

**Table 4:** Results of the hardness and tensile tests. Standard deviations are given in brackets next to the mean values.

Hardness and Tensile Tests Results	Ti–6Al–4V			Co–Cr–Mo		
-	HRC	UTS (MPa)	ε_b_ (%)	HRC	UTS (MPa)	ε_b_ (%)
X	39.8 (2.58)	1110 (1)	12.1 (1.5))	46.9 (1.13)	1282 (11)	12.8 (0.4)
XY	38.7 (3.85)	1098 (25)	12.7 (0.6)	46.9 (0.93)	1290 (6)	14.1 (0.2)
Y	38.9 (2.53)	1080 (5)	11.4 (0.9)	47.0 (0.76)	1300 (7)	12.9 (0.6)

**Table 5:** Tensile properties reported in literature for the Ti–6Al–4V and the Co–Cr–Mo alloys.

Ti–6Al–4V			Co–Cr–Mo		
AM Process	UTS (MPa)	ε_b_ (%)	AM Process	UTS (MPa)	ε_b_ (%)
Minimum [31]	896	4	L-PBF [32]	912	10.7
EBM [33]	946	13.2	L-PBF [34]	817	10.5
L-PBF [35]	1250	7	-	-	-
L-PBF + HIP [33]	997	11.4	-	-	-

**Table 6:** Values resulting from the *t*-test for the variables HRC, UTS, ε_b_ among the groups built in different orientations. Records below the level of significance of 0.05 are bold.

*p*-Values	Ti–6Al–4V			Co–Cr–Mo		
-	HRC	UTS (MPa)	ε_b_ (%)	HRC	UTS (MPa)	ε_b_ (%)
X vs. XY	0.29	0.31	0.39	0.76	0.18	0.37
X vs. Y	0.26	0.00	0.66	0.91	0.01	0.38
XY vs. Y	0.86	0.16	0.75	0.62	0.05	0.46
